# Symptomatic Pheochromocytoma: A Risk Model

**DOI:** 10.3390/cancers18030528

**Published:** 2026-02-06

**Authors:** María Consuelo Muñoz, Beatriz Febrero, Miriam Abellán, José Manuel Rodríguez

**Affiliations:** 1 Hospital Comarcal del Noroeste, 30400 Murcia, Spain; 2 Hospital Clínico Universitario Virgen de la Arrixaca, 30120 Murcia, Spain; miriam.abellan2@carm.es (M.A.); jmrodri@um.es (J.M.R.); 3 Department of Surgery, Pediatrics, Obstetrics and Gynecology, University of Murcia, 30100 Murcia, Spain; 4 Murcian Institute for Biomedical Research (IMIB) Pascual Parrilla, 30120 Murcia, Spain

**Keywords:** Pheochromocytoma, risk model, symptoms

## Abstract

**Background/Objectives**: Pheochromocytoma (PHEO) is increasingly detected incidentally or through genetic screening; however, predictors of symptomatic presentation and its perioperative impact remain unclear. We aimed to quantify the prevalence of symptomatic PHEO, identify associated factors, compare perioperative outcomes, and develop a predictive nomogram. **Methods**: We retrospectively analyzed patients diagnosed and/or operated on for PHEO at a tertiary referral center between 1984 and 2021. Associations with symptomatic presentation were assessed using univariate and multivariable logistic regression analyses. A nomogram was constructed based on independent predictors and evaluated using receiver operating characteristic (ROC) analysis and the area under the curve (AUC). **Results**: Among 173 patients (mean age 44.4 ± 15.8 years; 53.2% women), 67.1% were symptomatic. In multivariable analysis, male sex (odds ratio [OR] 0.33; *p* = 0.023) and the presence of a germline mutation (OR 0.15; *p* = 0.004) were associated with a lower likelihood of symptoms, whereas a noradrenergic secretion profile was associated with a higher likelihood (OR 12.73; *p* = 0.02). Symptomatic patients had higher rates of intraoperative (OR 2.60; *p* = 0.032) and postoperative complications (OR 3.09; *p* = 0.04). The nomogram incorporating sex, genetic status, and noradrenergic profile demonstrated moderate discrimination (AUC 0.799; 95% confidence interval 0.722–0.877; *p* < 0.001). **Conclusions**: Symptomatic PHEO is associated with sex, genetic status, and noradrenergic secretion profile and is linked to increased perioperative morbidity. A simple nomogram based on readily available variables may help estimate individual risk and support perioperative management.

## Simple Summary

Pheochromocytoma is increasingly detected incidentally or through genetic screening, resulting in a growing proportion of patients without typical symptoms. We retrospectively studied 173 patients diagnosed and/or operated on for pheochromocytoma at a tertiary hospital between 1984 and 2021 to identify factors associated with symptomatic presentation and to compare perioperative outcomes. Approximately two-thirds of patients were symptomatic. Female sex, sporadic disease (negative genetic testing), and a noradrenergic secretion profile were independently associated with symptomatic presentation. Symptomatic patients experienced higher rates of intraoperative and postoperative complications. We also developed a nomogram based on sex, genetic status, and secretion profile to estimate an individual patient’s probability of being symptomatic, showing moderate discriminatory ability. This tool may assist clinicians in identifying higher-risk patients and optimizing preoperative evaluation and perioperative monitoring.

## 1. Introduction

Pheochromocytoma (PHEO) is a low-prevalence tumor; however, its diagnosis has increased in recent years due to changes in diagnostic strategies and patterns of presentation [1]. Traditionally, diagnosis has relied on the patient’s clinical presentation, which is often nonspecific, leading to diagnostic delays of several years [2,3] or, in some cases, detection only at post-mortem examination [4]. New diagnostic approaches include imaging studies, such as adrenal incidentalomas, and family screening when a pathogenic mutation is known in advance. These strategies have led to the identification of asymptomatic PHEO and have helped ensure that patients with very mild or nonspecific clinical manifestations, even in the absence of overt symptoms, are not overlooked [5].

Diagnosis of PHEO as an adrenal incidentaloma has shown the greatest increase. Several studies support this observation. Berends et al. reported that the proportion of PHEO diagnosed through imaging studies doubled between 1995 and 2015 [6], and Gruber et al. further confirmed this trend, reporting imaging-based diagnosis in up to 62% of cases and identifying it as the most common mode of detection [7]. Patients diagnosed incidentally are typically older and less symptomatic, and are often entirely asymptomatic [6,8].

Given the strong association between PHEO and genetic predisposition, genetic testing is increasingly performed both in patients with PHEO and in relatives of individuals carrying germline mutations, leading to the identification of new mutation carriers. Consequently, family screening now accounts for a substantial proportion of PHEO diagnoses [6,9,10,11].

As a result of these medical advances, the incidence of PHEO has increased nearly fivefold over the past four decades. A key aspect of this diagnostic evolution is that tumors are increasingly identified at asymptomatic or minimally symptomatic stages, which is associated with improved short- and long-term prognosis. Early detection reduces the high cardiovascular risk associated with this tumor, as well as tumor-related and perioperative mortality.

## 2. Materials and Methods

### 2.1. Patients and Definitions

Retrospective study of patients operated on and/or diagnosed with PHEO in a tertiary hospital between 1984 and 2021.

Inclusion criteria were histological confirmation of PHEO and/or biochemical and radiological diagnosis (both) and a complete clinical history. We considered as PHEO both those diagnosed by biochemistry and imaging tests and those diagnosed only by histological examination.

The biochemical diagnosis was carried out by 24-h urinary determination of catecholamines (adrenaline, noradrenaline and dopamine), metanephrines, normetanephrines and vanillyl mandelic acid. To confirm the biochemical diagnosis, an imaging test was performed, usually Computed Tomography (CT) or Magnetic Resonance Imaging (MRI), and where necessary, nuclear imaging techniques such as 123-metaiodobenzylguanidine (123-MIBG) or 68-Gallium-DOTATOC (68 Ga-DOTATOC) have been used. In case of adrenal incidentaloma, the biochemical diagnosis was subsequent to the imaging diagnosis.

### 2.2. Variables

The variables analysed were epidemiological (age and sex), type of diagnosis (imaging, screening or symptomatology), laterality and bilaterality of the lesion, asymptomatic or symptomatic patients (including type of clinical manifestation), genetics, catecholaminergic profile, histology (tumour size) and intraoperative and postoperative complications.

### 2.3. Statistical Analysis

Basic descriptive methods were employed to analyse the sample. For qualitative variables, the number of cases in each category and their respective percentages were obtained. For quantitative variables, the minimum, maximum, mean and standard deviation values were obtained.

The logistic regression model (univariate and multivariate) was used to determine the effect of demographic variables, location, biochemical and histological data in association with patients with symptomatic PHEO. Similarly, the effect of being symptomatic or not was also assessed in relation to intraoperative and postoperative complications. Multivariate models were evaluated using the Hosmer and Lemeshow test and the league table. Statistical analysis was performed using SPSS 28.0 for Windows. Differences with a *p* < 0.05 were considered statistically significant.

A predictive nomogram was developed using the statistically significant variables from the multivariate models of symptomatic PHEO. The AUC was assessed using the Swets criterion.

## 3. Results

### 3.1. Overall Description of the Series

A total of 173 patients were included. The mean age at diagnosis was 44.36 ± 15.80 years, and ninety-two patients were female (53.18%). The diagnosis was made clinically in 48.55% (n = 84), by genetic screening in 36.42% (n = 63), and as an adrenal incidentaloma in 15.03% (n = 26). 39.31% were bilateral (n = 68), and of all PHEO, one hundred and ten were found in the right gland (49.55%) and one hundred and twelve in the left gland (50.45%). 64.71% (n = 88) of patients who underwent genetic testing had a mutation.

32.95% (n = 57) of patients were asymptomatic, 67.05% (n = 116) presented symptoms, 84 presented with clinical manifestations and the remaining 32 (diagnosed as adrenal incidentaloma or by genetic screening) presented signs and/or symptoms suggestive of PHEO in the medical history. Symptoms were categorized as follows: hypertension (49.71%, n = 86), cardiac manifestations (34.10%, n = 59), neurological symptoms (31.79%, n = 55), skin manifestations (27.17%, n = 47), systemic symptoms (24.28%, n = 42), digestive symptoms (19.65%, n = 34), psychiatric symptoms (12.14%, n = 21), renal manifestations (9.25%, n = 16), pulmonary symptoms (8.67%, n = 15), vascular disease (6.36%, n = 11), the classic triad (6.36%, n = 11), obstetric manifestations (1.16%, n = 2), and ophthalmological manifestations (1.16%, n = 2). With regard to the catecholaminergic secretion profile, the most frequent profile was mixed (54.29%), followed by adrenergic (23.57%), noradrenergic (20%) and, finally, dopaminergic (2.14%).

In terms of size, the mean size of the PHEO was 4.07 ± 3.08 cm.

A total of 154 patients (93.33%) received preoperative medical treatment. The most commonly used agent was phenoxybenzamine (83.61%, n = 138), followed by doxazosin (18.18%, n = 28); 8.44% of patients (n = 13) received both agents. After adequate α-blockade, β-blockers were initiated in 12.99% of patients (n = 20).

Eight patients were not operated on due to either metastatic disease with extensive tumor burden or high surgical risk related to comorbidities. Consequently, 165 patients (95.38%) underwent surgery. Of these, 70.91% (n = 117) underwent unilateral adrenalectomy, while 29.09% (n = 48) underwent bilateral adrenalectomy during the same surgical procedure.

Surgical approach was laparoscopic in 60.6% of cases (n = 100), with conversion to open surgery required in 10% (n = 10). Open surgery was performed as the primary approach in 39.39% of patients (n = 65).

Surgical complications occurred in 23.03% of procedures (n = 38). Among intraoperative complications, hypertension was the most frequent, reported in 39.47% of cases (n = 15), followed by bleeding in 26.32% (n = 10). Other complications were observed less frequently.

Postoperative complications were recorded in 15.76% of patients (n = 26). The most common was fever, affecting 38.46% of patients with postoperative complications (n = 10), followed by hypotension in 34.61% (n = 9) and hypertension in 19.23% (n = 5).

### 3.2. Differences Between Patients with Symptomatic and Asymptomatic PHEO: Profile of Patients with Symptomatic PHEO (Table 1)

With regard to gender, symptomatic PHEO was more common in women than in men (76.1% vs. 58%). Men were 2.32 times less likely to have symptoms than women (1/OR = 2.32; *p* = 0.012).

In terms of age, the mean age at diagnosis in symptomatic patients was 45.92 ± 16.03 years and in asymptomatic patients 41.31 ± 15.13 years, with no statistical significance.

The laterality of the tumor did not influence whether patients presented with symptoms. However, the study assessed whether the presence of synchronous bilateral PHEO influenced the likelihood of presenting symptoms and found that synchronous bilateral PHEO reduced the likelihood of symptoms by 3.7 times (1/OR = 3.7; *p* < 0.001).

In terms of genetics, the 136 patients who had undergone genetic testing were included. A positive genetic test result was statistically significantly associated with having an asymptomatic PHEO, with a 10-fold lower probability of presenting symptoms (1/OR = 10; *p* < 0.001).

Regarding the catecholaminergic profile, the mixed, noradrenergic and adrenergic profiles were statistically significant. The noradrenergic and mixed profiles were associated with a 3.63-fold (OR = 3.63; *p* = 0.017) and a 2.09-fold (OR = 2.09; *p* = 0.029) increase in the probability of symptoms, respectively. The adrenergic profile was the most prevalent among asymptomatic patients (57.6%), and the univariate analysis revealed that it reduced the likelihood of exhibiting symptoms by 3.57-fold (*p* = 0.001).

There was also a statistically significant relationship between size and having symptomatic PHEO. Symptomatic cases showed larger sizes, with a mean size of 4.49 ± 3.07 cm, compared to 3.14 ± 2.27 cm in asymptomatic cases. Larger size increased the probability of being symptomatic by 1.24 times (OR = 1.24; *p* = 0.006).

**Table 1:** Differences between symptomatic and asymptomatic PHEO. Univariate analysis.

Variable	Asymptomatic	Symptomatic	OR (IC 95%)	*p*-Value
Female sex	22 (23.9%)	70 (76.1%)	1	
Male sex	34 (42%)	47 (58%)	0.43 (0.23–0.83)	**0.012**
Age	41.31 ± 15.13 years	45.92 ± 16.03 years	1.02 (0.99–1.04)	0.074
Laterality				
Right	27 (24.6%)	83 (75.4%)	1	
Left	27 (24.2%)	85 (75.8%)	0.98 (0.43–2.23)	0.959
Bilateralism				
No	26 (24.8%)	79 (75.2%)	1	
Yes	37 (55.1%)	31 (44.9%)	0.27 (0.14–0.54)	**<0.001**
Genetic				
No	5 (10.4%)	43 (89.6%)	1	
Yes	47 (53.4%)	41 (46.6%)	0.10 (0.04–0.28)	**<0.001**
Adrenergic profile				
No	37 (27.8%)	96 (72.2%)	1	
Yes	19 (57.6%)	14 (42.4%)	0.28 (0.13–0.63)	**0.001**
Noradrenergic profile				
No	52 (37.7%)	86 (62.3%)	1	
Yes	4 (14.3%)	24 (85.7%)	3.63 (1.19–11.04)	**0.001**
Dopaminergic profile				
No	55 (33.7%)	108 (66.3%)	1	
Yes	1 (33.3%)	2 (66.7%)	1.02 (0.09–11.48)	0.988
Mixed profile				
No	37 (41.1%)	53 (58.9%)	1	
Yes	19 (25%)	57 (75%)	2.09 (1.07–4.08)	**0.029**
Size	3.14 ± 2.27 cm	4.49 ± 3.07 cm	1.24 (1.06–1.44)	**0.006**

Bold font indicates *p *< 0.05: statistically significant values.

### 3.3. Influence of Symptomatic PHEO on Intraoperative and Postoperative Complications

We analysed whether the presence of symptoms was associated with intraoperative and postoperative complications. Intraoperative complications were more frequent in symptomatic patients, and we found a statistically significant relationship, with symptomatic patients being 2.6 times more likely to experience them (OR = 2.60; *p* = 0.032). The same was true for postoperative complications, which were more prevalent in symptomatic patients. There was a statistically significant relationship, with symptomatic patients being 3.09 times more likely to experience postoperative complications (OR = 3.09; *p* = 0.04) (Table 2 and Table 3).

### 3.4. Differences Between Symptomatic and Asymptomatic PHEO: Multivariate Analysis

Table 4 shows the results of the multivariable logistic regression performed to determine the effect of the variables that were significant in the previous univariate analysis on the prediction of symptomatic presentation.

Variables that remained statistically significant were sex, positive genetic testing, and a noradrenergic secretion profile, maintaining the effects observed in the univariate analysis. Male sex and positive genetic testing were associated with a lower likelihood of being symptomatic (1/OR = 3.03; *p* = 0.023 and 1/OR = 6.67; *p* = 0.004, respectively), whereas a noradrenergic secretion profile was associated with a higher likelihood of symptoms (OR = 12.73; *p* = 0.02). In addition, symptomatic patients had a higher likelihood of intraoperative complications (OR = 5.34; *p* = 0.021).

### 3.5. Development of the Predictive Model and Predictive Nomogram (Risk Calculator)

A predictive nomogram was developed using statistically significant variables from the multivariate models of the variables that influenced the presentation of symptomatic PHEO (Appendix A).

The following formula was used to calculate the probability of presenting familial PHEO, using the estimated statistical predictive model:

*p* = 1/(1 + e^(−(alpha + beta_1 × x_1 + … + beta_n × x_n)))

where

α is the constant;
x1… xn are the variables presented in the model.

Once the probability was calculated, it was determined that:

If *p* ≤ 0.5: Asymtomatic PHEO;
If *p* > 0.5: Symtomatic PHEO.

Therefore, the formula for the model is:

*p* = 1/(1 + e^(−a))

a = −(2.35 − 0.92 × Sex (Male = 1, Female = 0) − 2.23 × Positive genetics (Yes = 1, No = 0) +1.51 × Noradrenergic secretion profile (Yes = 1, No = 0))

On the other hand, the diagnostic validity index values of the model were high (Table 5).

A nomogram was created using significant predictive variables to determine the probability of symptomatic PHEO. To validate the nomogram, we calculated the area under the curve (AUC) of the receiver operating characteristic (ROC) curve.

**Figure 1:**
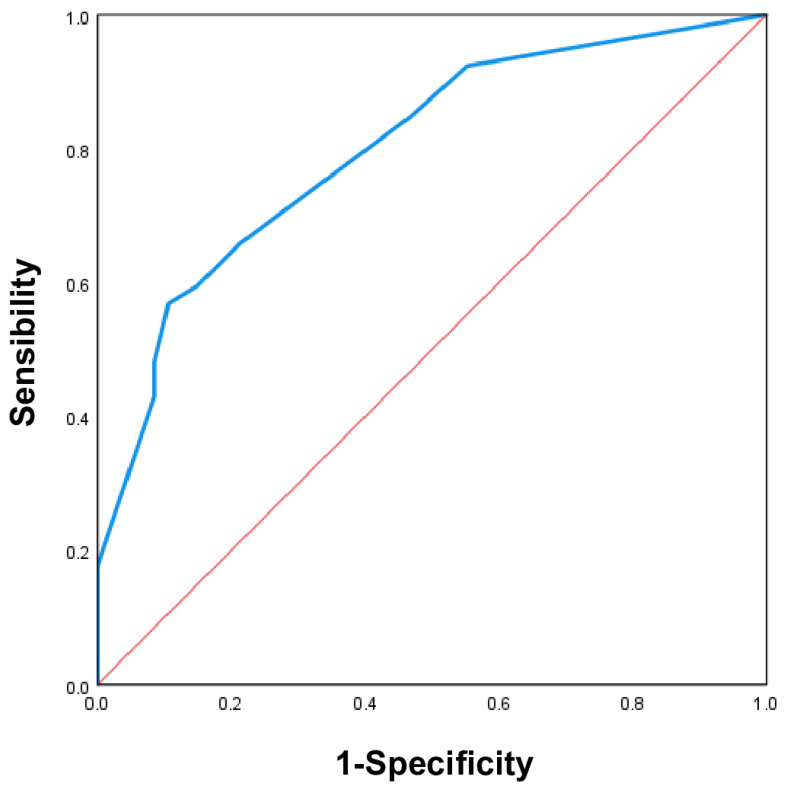
ROC curve. Area under the curve (AUC) of the ROC (receiver operating characteristic) curve.

The value was found to be 0.799 (95% CI: 0.722–0.877; *p* < 0.001), which indicates moderate accuracy and, therefore, moderate validity of the nomogram. This can be seen in Figure 1.

## 4. Discussion

The mode of diagnosis of pheochromocytoma (PHEO) has changed substantially over recent decades, with a marked increase in diagnoses made through adrenal incidentalomas and genetic screening [1]. As a result, a growing proportion of patients with PHEO do not present with overt clinical symptoms. Mannelli et al. described these cases as subclinical or asymptomatic PHEO [12].

Early detection of PHEO in asymptomatic stages is clinically relevant, as failure to diagnose this tumor is associated with significant morbidity and mortality [13]. Although asymptomatic PHEO appears to carry a lower cardiovascular risk than symptomatic disease, some patients may still experience adverse outcomes due to continuous catecholamine secretion [14,15].

With regard to sex differences, women showed a higher prevalence of symptomatic PHEO than men, a finding that was statistically significant in both univariate and multivariate analyses. Edwin et al. similarly reported that women exhibited more signs and symptoms of PHEO than men, independent of biochemical phenotype and tumor presentation, and suggested that sex-related differences in catecholamine receptor sensitivity may play a role [16].

A trend toward a lower mean age at diagnosis was observed among asymptomatic patients, although this did not reach statistical significance. This finding is likely explained by the high proportion of patients diagnosed through genetic screening, who are typically identified at younger ages. In contrast, PHEO diagnosed as adrenal incidentalomas tends to occur later in life [17,18].

Our analysis showed that the presence of synchronous bilateral PHEO was associated with a lower likelihood of symptomatic presentation in univariate analysis; however, this association did not persist in the multivariate model. This finding is likely attributable to the high prevalence of RET mutations associated with multiple endocrine neoplasia type 2A (MEN 2A) in our cohort, as these patients are often diagnosed through screening before symptoms develop, despite having bilateral disease.

From a genetic perspective, familial cases were significantly less symptomatic than sporadic cases in both univariate and multivariate analyses. These findings are consistent with those of Neumann et al., who demonstrated that asymptomatic PHEO is increasingly identified through genetic screening programs [9].

Regarding catecholaminergic secretion profiles, mixed and noradrenergic profiles were associated with a higher probability of symptomatic PHEO in univariate analysis, whereas an adrenergic profile appeared to be protective. In multivariate analysis, only the noradrenergic profile remained independently associated with symptomatic presentation. The literature on this topic is heterogeneous and sometimes contradictory. Traditionally, noradrenergic PHEO—often associated with cluster 1 tumors—has been considered less symptomatic, with milder and less paroxysmal clinical manifestations [19,20]. These tumors are frequently associated with sustained rather than paroxysmal hypertension, resembling essential hypertension [21]. Zuber et al. linked sustained hypertension to continuously elevated catecholamine levels, suggesting a persistent rather than episodic secretory pattern [22]. Chronic exposure to high noradrenaline concentrations may also induce adrenergic receptor desensitization, further attenuating clinical manifestations [12,22,23].

Despite these considerations, our results indicate that a noradrenergic secretion profile is associated with a higher likelihood of symptomatic disease. Falhammar et al. similarly reported a positive correlation between noradrenergic secretion and symptom burden [24]. Although specific symptom patterns were not analyzed in detail in our study, these manifestations may be more subtle and sustained rather than overtly paroxysmal.

The adrenergic secretion profile, characteristic of cluster 2 tumors, is typically associated with paroxysmal and more striking symptoms that may be triggered by specific stimuli [25]. However, these tumors may remain asymptomatic in the absence of triggering events, which could explain their higher prevalence among asymptomatic patients in our cohort. Additionally, the high proportion of MEN 2A cases diagnosed through screening in our population likely contributed to early detection prior to symptom development [26].

An important limitation of this study is the incomplete availability of detailed information on regular medication use at the time of diagnosis, particularly antihypertensive therapy such as alpha- and beta-blockers. Given the long retrospective inclusion period, pharmacological treatment could not be systematically incorporated into the analysis. This is clinically relevant, as medical therapy may attenuate or mask symptoms related to catecholamine excess, potentially leading to misclassification of some patients as asymptomatic. Consequently, this factor may have influenced symptom-based stratification and should be considered when interpreting the results and the proposed risk model.

Tumor size was larger in symptomatic than in asymptomatic patients, and this association was statistically significant in univariate analysis but not in the multivariate model. These findings are consistent with those reported by Iglesias et al., who observed that larger tumors were more likely to be symptomatic, whereas smaller lesions were often asymptomatic [27].

A key contribution of our study is the development of a predictive nomogram to estimate the probability of symptomatic presentation in patients with PHEO. Unlike previous studies that primarily describe symptom patterns, this tool integrates readily available clinical variables—sex, genetic status, and catecholaminergic secretion profile—to generate an individualized risk estimate. This approach may help refine clinical decision-making, guide follow-up strategies, and support a more personalized management of PHEO.

We also evaluated whether symptomatic presentation was associated with increased intraoperative and postoperative complications. Univariate analysis revealed a significant association with both outcomes, with intraoperative complications remaining significant in the multivariate model. Data addressing the relationship between symptom burden and perioperative complications are limited. Li et al. evaluated risk factors for hemodynamic instability during surgery for PHEO larger than 5 cm and included symptom status, although no significant association was observed [28]. Jiang et al. analyzed preoperative hypertension, hyperglycemia/diabetes mellitus, and tachycardia in relation to perioperative complications, identifying hyperglycemia/diabetes mellitus as the only significant predictor [29]; however, it was unclear whether these conditions were pre-existing or directly related to PHEO. Araujo-Castro et al. assessed hypertension, diabetes mellitus, cardiovascular disease, and cerebrovascular disease, reporting significant associations for diabetes mellitus and cerebrovascular disease, although the causal relationship with PHEO was not specified [30]. Beyond these studies, data on the relationship between symptomatology and intraoperative complications remain scarce.

Regarding postoperative complications, Araki et al. evaluated the risk of hypoglycemia and symptom burden but did not observe statistically significant associations [31]. In contrast, Nölting and Grossman reported significant associations between diabetes mellitus, coronary artery disease, hypertension, arrhythmias, and postoperative complications, although the extent to which these conditions were attributable to PHEO was not clarified [32].

## 5. Conclusions

Symptomatic pheochromocytoma is more prevalent among women and patients with sporadic disease, particularly those with a noradrenergic secretion profile. In addition, symptomatic patients are at increased risk of intraoperative complications.

## Figures and Tables

**Table 2:** Influence of symptomatic PHEO on intraoperative complications. Univariate analysis.

Variable	Intrasurgical Complications		OR (IC 95%)	*p*-Value
	No	Yes		
Symptomatic				
No	47 (87%)	7 (13%)	1	
Yes	81 (73%)	30 (27%)	0.43 (0.23–0.83)	**0.032**

Bold font indicates *p *< 0.05: statistically significant values.

**Table 3:** Influence of symptomatic PHEO on postoperative complications. Univariate analysis.

Variable	Postoperative Complications		OR (IC 95%)	*p*-Value
	No	Yes		
Symptomatic				
No	50 (92.6%)	4 (7.4%)	1	
Yes	89 (80.2%)	22 (19.8%)	3.09 (1.01–9.47)	**0.04**

Bold font indicates *p *< 0.05: statistically significant values.

**Table 4:** Differences between symptomatic and asymptomatic PHEO: Multivariate analysis.

Variable	OR (IC 95%)	*p*-Value
Sex	0.33 (0.13–0.86)	**0.023**
Bilateralism	1.73 (0.61–4.95)	0.305
Positive genetic test	0.15 (0.04–0.54)	**0.004**
Adrenergic profile	0.87 (0.2–3.78)	0.857
Noradrenergic profile	12.73 (1.91–85.06)	**0.02**
Mixed profile	2.94 (0.79–10.97)	0.108
Size	1.04 (0.81–1.33)	0.747
Intraoperative complications	5.34 (1.29–22.16)	**0.021**
Postoperative complications	1.52 (0.28–8.41)	0.631

Bold font indicates *p *< 0.05: statistically significant values.

**Table 5:** Validation indices of the predictive model’s diagnostic capacity.

Index % (IC 95%)				
	Sensibility	Specificity	PPV	NPV
Model	84.8 (76.3–93.4)	53.2 (37.9–68)	75.3 (65.8–84.8)	67.6 (51.1–84)

PPV (positive predictive value); NPV (negative predictive value).

**Figure A1:**
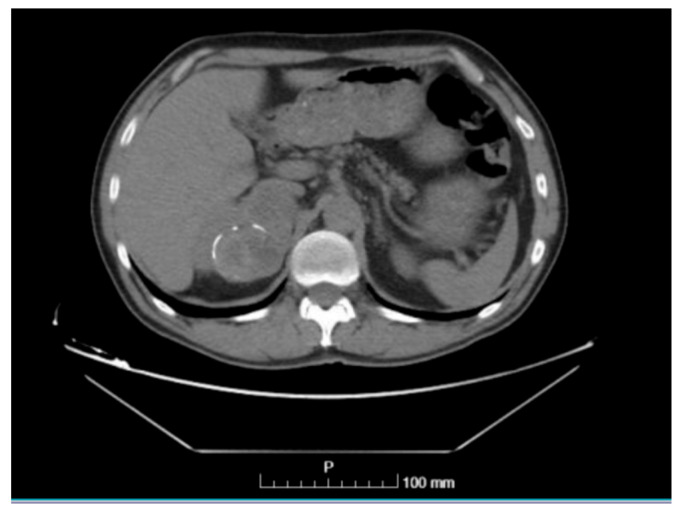
CT image that shows a right adrenal mass (up to 8.7 cm maximum diameter), with lobulated edges and heterogeneous density, with areas of cystic/necrotic degeneration and linear calcifications inside. The lesion shows contact with the posterior slope of the right posterior hepatic segments (s6–s7), the posterior surface of the inferior vena cava and the entrance to the right renal vein, as well as the right diaphragmatic pillar. No signs of vascular thrombosis are identified. Left adrenal gland without alterations.

**Figure A2:**
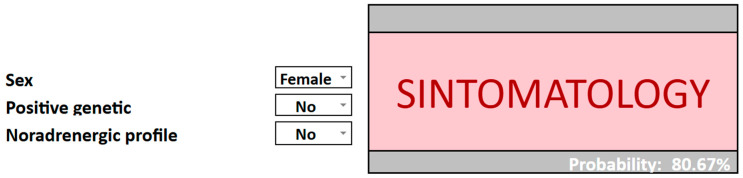
Predictive nomogram that includes sex, genetic test result and secretion profile as predictive variables for symptomatic pheochromocytoma. The pheochromocytoma of this patient has an 80% probability of presenting symptoms.

## Data Availability

The data presented in this study are available on request from the corresponding author. The data are not publicly available due to privacy and ethical restrictions related to patient information.

## Abbreviations

The following abbreviations are used in this manuscript: AUC, area under the curve; BP, blood pressure; CI, confidence interval; CT, computed tomography; CV, cardiovascular; DM, diabetes mellitus; MRI, magnetic resonance imaging; MIBG, metaiodobenzylguanidine; NPV, negative predictive value; OR, odds ratio; PHEO, pheochromocytoma; PPV, positive predictive value; ROC, receiver operating characteristic; SD, standard deviation; SPSS, Statistical Package for the Social Sciences; VMA, vanillylmandelic acid; ^123^I-MIBG, iodine-123 metaiodobenzylguanidine; ^68^Ga-DOTATOC, gallium-68 DOTATOC.

## Appendix A. Case Example

A 45-year-old woman with a history of palpitations and sweating is admitted to the emergency department for a hypertensive crisis. The patient reports high blood pressure prior to admission. An abdominal CT scan is performed as an imaging test, revealing a right adrenal tumour (Figure A1). 24 h urinary cathecolamines and metanephrines values were requested two times and found to be elevated (more than twice the upper value), the results were: noradrenaline 3023 µg/24 h (12–86), adrenaline 5028 µg/24 h (2–23), dopamine 399 µg/24 h (80–600), normetanefrine 4840 µg/24 h (120–650), metanefrine 10,850 µg/24 h (60–350). With a diagnosis of pheochromocytoma, genetic test was requested, with negative result. A predictive nomogram was calculated using statistically significant variables that influenced the presentation of symptomatic PHEO (Figure A2).
